# An accessory sphenoidal foramen of the middle cranial fossa detected on computed tomography

**DOI:** 10.1007/s00276-025-03601-3

**Published:** 2025-03-10

**Authors:** George Triantafyllou, Panagiotis Papadopoulos-Manolarakis, Łukasz Olewnik, Fabrice Duparc, George Tsakotos, Nicol Zielinska, Maria Piagkou

**Affiliations:** 1 Department of Anatomy, School of Medicine, Faculty of Health Sciences, National and Kapodistrian University of Athens, 75 Mikras Asias Str., Goudi, 11527 Athens, Greece; 2 Department of Neurosurgery, General Hospital of Nikaia-Piraeus, Athens, Greece; 3 Department of Clinical Anatomy, Masovian Academy in Płock, Płock, Poland; 4 Department of Anatomy, Faculty of Medicine-Pharmacy, University of Rouen-Normandy, Rouen, France

**Keywords:** Middle cranial fossa, Accessory foramen, Variant foramen, Emissary foramen, Infratemporal fossa, Anatomy, Variation

## Abstract

**Background:**

The skull base depicts significant morphological variability, which is frequently described due to its neurosurgical significance. The middle cranial fossa's accessory foramen has rarely been described.

**Materials:**

A 53-year-old female patient's computed tomography (CT) scan was further investigated for its unusual morphology.

**Results:**

On the left-sided middle cranial fossa, an accessory sphenoidal foramen (ASF) was observed, located 3.3 mm posterior to the foramen rotundum (FR) and 5.5 mm anterior to the foramen ovale (FO). Extracranially, the ASF opened into the infratemporal fossa and coexisted with another sphenoidal emissary foramen (SEF), anteromedially to the FO. On the right side, two SEF were located anteromedially to the FO.

**Conclusions:**

Similar to the current case, ASF of the middle cranial fossa were reported in a previous study with a prevalence of 0.20%. The unconstraint well described accessory foramina are the emissary foramina that transmit emissary veins, and are of interest for anatomists, radiologists and neurosurgeons.

## Introduction

The skull base exhibits exciting morphological variability with considerable neurosurgical interest [2, 3, 7]. It is typically divided into the anterior, middle, and posterior cranial fossae for a detailed investigation [11].

The sphenoid and temporal bones form the middle cranial fossa. Three constant foramina can be identified in the sphenoid bone’s greater wing. The foramen rotundum (FR) is located posteriorly to the superior orbital fissure medial end and opens into the pterygopalatine fossa. The FR usually transmits the maxillary nerve (V2). The foramen ovale (FO) is typically seen behind the FR and contains the mandibular nerve (V3). Finally, the foramen spinosum (FS) is posterolateral to the FO and transmits the middle meningeal artery and veins. Both FO and FS open into the infratemporal fossa (ITF) [11]. The morphological variability of these constant structures has been well reported, as well as emissary foramina that can transmit emissary veins, such as the sphenoidal emissary foramen (SEF) [3–5].

Herein, we report the rare middle cranial fossa accessory sphenoidal foramen (ASF) variant detected incidentally during computed tomography scan (CT).

## Case report

An unusual variant of ASF was identified during a retrospective skull CT investigation of a Greek adult population sample. Due to its uniqueness, the skull of a 53-year-old female patient was further investigated at the middle cranial fossa by performing a sinus CT (0.6 mm thickness). The investigation was conducted and documented using the Horos software (Horos Project) and 3D Slicer. Evidence was obtained on the multiplanar reconstruction of the axial, coronal, and sagittal slices and their three-dimensional volume reconstruction.

**Fig. 1:**
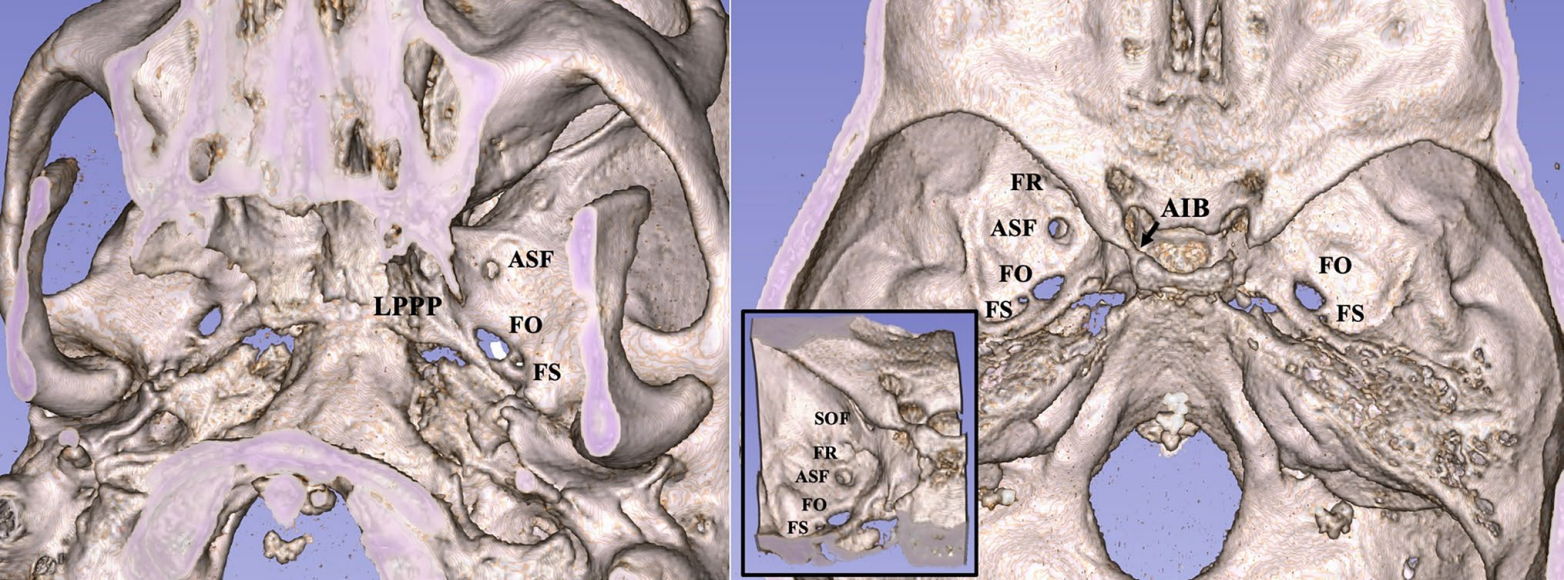
The computed tomography depicts the accessory sphenoidal foramen (ASF) of the middle cranial fossa. Three-dimensional reconstruction of the CT, left figure: extracranial view of the middle cranial fossa depicting the ASF opening into the infratemporal fossa. Right figure: intracranial view. Small figure: intracranial view with the superior orbital fissure (SOF). *FO* foramen ovale, *FS* foramen spinosum, *SEF* sphenoidal emissary foramen, *FR* foramen rotundum, *AIB* anterior interclinoid bar

On the left-sided middle cranial fossa, the FO (of anteroposterior and lateromedial diameters 6.7 mm and 3.6 mm) was observed as typical. The FS (of anteroposterior and laterolateral diameters of 1.7 mm and 0.8 mm) was typically found posterolateral to the FO. Anterior to the FO (distance of 5.5 mm), a variant ASF of a round shape was detected. Its anteroposterior and laterolateral diameters were 4 mm and 3.4 mm. The ASF was 3.3 mm posteriorly to the FR and opened extracranially to the ITF. It was located at a distance 3.6 mm lateral to the tip of the lateral pterygoid plate. A SEF coexisted anteromedially with the FO (Fig. 1). In addition, another ossified variant was observed, this of the complete ossified anterior interclinoid ligament, connecting the anterior and posterior clinoid processes.

**Fig. 2:**
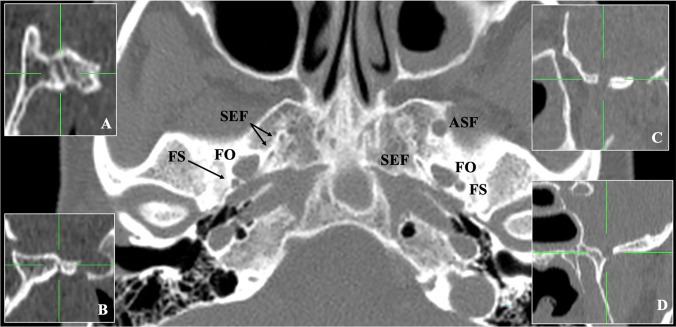
The computed tomography depicts the accessory sphenoidal foramen (ASF) of the middle cranial fossa. Axial plane. *FO* foramen ovale, *FS* foramen spinosum, *SEF* sphenoidal emissary foramen. **A** Sagittal plane of the first right SEF, **B** sagittal plane of the second right SEF, **C** sagittal plane of the ASF, **D** coronal plane of the ASF

On the right side, the FO and FS were observed as typical. The variant ASF was not identified. Two SEF were identified anteromedially to the FO (Fig. 2).

## Discussion

The variant ASF was incidentally detected during a retrospective imaging study of 168 CT scans. The prevalence was estimated at 0.60% per skull or 0.30% per side. Such an ASF was not observed in our osteological collection comprised of 120 adult dried skulls. Based on the location of the variant ASF, closer to the FR and having exit at the ITF, we can hypothesize that the content could be accessory branches of the middle meningeal artery, or the pterygomeningeal artery [10], as well as emissary veins from the pterygoid venous plexus entering the skull base and anastomose with the cavernous sinus area. The bilateral coexistence of SEF also justifies this possibility. Another possibility based on Rusu's [8] dissection report is a possible FO duplication, with the ASF (accessory anteriorly located FO) containing the anterior division (trunk) of the mandibular nerve (V3), i.e., the branches of the pterygoid, masseteric, and temporalis muscles (motor component), as well as the buccal nerve (sensory) component. Thus, we hypothesize the probability of having a V3 higher division than usual. Another rare accessory foramen of the middle cranial fossa corresponds to the FR duplication. Rusu [8] identified an accessory division of the maxillary nerve (V2) entering this accessory FR, while the typical V2 transmitted through the typical FR. It is essential to highlight that the accessory FR was located posteriorly to the typical one [8]. Syed et al. [12] reported another accessory FR case while investigating a dried adult skull. This variant foramen was lateral to the typical FR, contrary to Rusu's [8] case. They suggested four possible contents of the accessory FR: split part of the maxillary nerve, emissary veins of the FR interconnecting the cavernous sinus with the veins below the skull base, artery of the FR, or aberrant course of the Vidian nerve [12]. Hence, these two case reports reported actual duplication of the FR [8, 12].

Variant accessory foramina of the middle cranial fossa have been previously described. Mazengenya and Ekpo [2] presented five out of 512 skulls with unilateral variants (0.98% prevalence). Four foramina were positioned anterolateral to the FR, while only one case was posterolateral. However, after inserting probes, they identified that only two skulls had “true foramina” while the other three were blind and shallow. The one case that was similar to the current report corresponded to the foramen posterolateral to the FR with a prevalence of 0.20% [2]. Raz et al. [6] reported a case of a dried skull, with a variant accessory foramen observed bilaterally. This case was dissimilar to the current one because they were identified 31 mm laterally to the FR and FO, contrary to our case, which was in the same plane (anteroposterior axis) as the FR and FO. These variant foramina were relatively small, with 0.82 mm and 0.7 mm diameter, respectively [6]. To the authors’ knowledge, these variant foramina were not identified in the old anatomical textbooks [1, 13].

In the current case, we identified bilateral SEF (one SEF on the left and two SEF on the right side) thus the extracranial pterygoid venous plexus interconnection with the cavernous sinus is justified. The SEF is a well-described variant with a pooled prevalence of 38.1% [5]. This foramen transmits a sphenoidal emissary vein interconnecting the pterygoid venous plexus to the cavernous sinus. [5]. Natsis et al. [3] investigated the influence of the SEF presence on the FO morphometry; they did not depict any significant association. They identified two skulls with SEF duplication, similar to the current case, with a prevalence of 1.03% per skull and 0.52%. Thus, the SEF duplication can be considered an infrequent variant. Peper et al. [4] presented a report of a dried adult skull with an enlarged SEF (4.34 mm diameter). Rusu [9], during an imaging investigation (computed tomography angiography), identified an exciting emissary foramen. A sphenopterygoid canal opened into a pterygoid foramen on the lateral pterygoid plate, that contained an emissary vein connecting the cavernous sinus with the pterygoid venous plexus [9].

To conclude, we reported a variant ASF located posteriorly to the FR and anteriorly to the FO opened into the ITF. Although their presence may be sporadic, knowledge of such variants is essential to consider before skull base procedures (with CT imaging). The sphenoidal emissary veins are surgically important, and neurosurgeons should keep in mind that the SEF is not the only passage [9].

## Data Availability

Please contact the authors for data requests (George Triantafyllou—email address: georgerose406@gmail.com).
